# A Case of Lymphomatoid Papulosis With CD15- and CD30-Positive Reed-Sternberg-Like Cells in CD8-Positive Mycosis Fungoides: Differential Diagnosis of Secondary Hodgkin Lymphoma and Large Cell Transformation of Mycosis Fungoides

**DOI:** 10.7759/cureus.98884

**Published:** 2025-12-10

**Authors:** Tomoki Sakiyama, Tomoyuki Shirase, Kodai Furuta

**Affiliations:** 1 Department of Dermatology, Kyoto University Graduate School of Medicine, Kyoto, JPN; 2 Department of Dermatology, Japanese Red Cross Otsu Hospital, Shiga, JPN; 3 Department of Pathology and Diagnosis, Japanese Red Cross Otsu Hospital, Shiga, JPN; 4 Department of Dermatology, Tenri Hospital, Nara, JPN

**Keywords:** cd15, cd30, lymphomatoid papulosis, mycosis fungoides, reed-sternberg-like cell

## Abstract

Mycosis fungoides (MF) is the most common type of cutaneous T-cell lymphoma, and cases with large cell transformation (LCT) are generally associated with a poor prognosis. Lymphomatoid papulosis (LyP) is a cluster of differentiation (CD)30-positive lymphoproliferative disorder characterized by recurrent eruptions and spontaneous regression, typically following a benign clinical course. Histopathologically, LyP occasionally contains Reed-Sternberg (RS)-like large cells, making it important to distinguish LyP from cutaneous involvement of Hodgkin lymphoma (HL) and MF with LCT. We report a case in which erythematous papules developed within MF lesions. Skin biopsy of the papule revealed RS-like large cells positive for CD15 and CD30. No abnormalities were detected by imaging studies or flow cytometry, and the papules regressed spontaneously, leading to a diagnosis of LyP. Treatment with oral etretinate and narrowband ultraviolet B phototherapy resulted in improvement of the MF lesions, and no recurrence of the erythematous papules has been observed. As LyP may morphologically resemble cutaneous involvement of HL or MF with LCT, comprehensive evaluation, including clinical course, imaging findings, and immunophenotypic analysis, is essential for accurate diagnosis.

## Introduction

Mycosis fungoides (MF) represents the most prevalent subtype of cutaneous T-cell lymphoma. With disease progression, it may evolve into tumor-stage lesions and undergo large cell transformation (LCT), a process frequently accompanied by the emergence of cluster of differentiation (CD)30-positive cells, which is associated with an adverse prognosis [1]. In contrast, lymphomatoid papulosis (LyP) is a CD30-positive cutaneous lymphoproliferative disorder that typically demonstrates an indolent clinical course with recurrent spontaneous regression. Histopathologically, LyP often harbors large Reed-Sternberg (RS)-like cells, which may closely mimic cutaneous involvement of Hodgkin lymphoma (HL) or MF with LCT, thereby complicating the differential diagnosis [2]. Expression of CD15 in addition to CD30 is rare in LyP [3], and such cases are particularly prone to diagnostic ambiguity.

This report describes a case in which papular lesions containing CD30- and CD15-positive RS-like cells developed within MF lesions and underwent spontaneous regression, ultimately diagnosed as LyP based on the clinical course and systemic findings. The clinical course and histopathological features are presented, with emphasis on the diagnostic challenges in distinguishing this entity from cutaneous involvement of HL and MF with LCT.

## Case presentation

**Figure 1:**
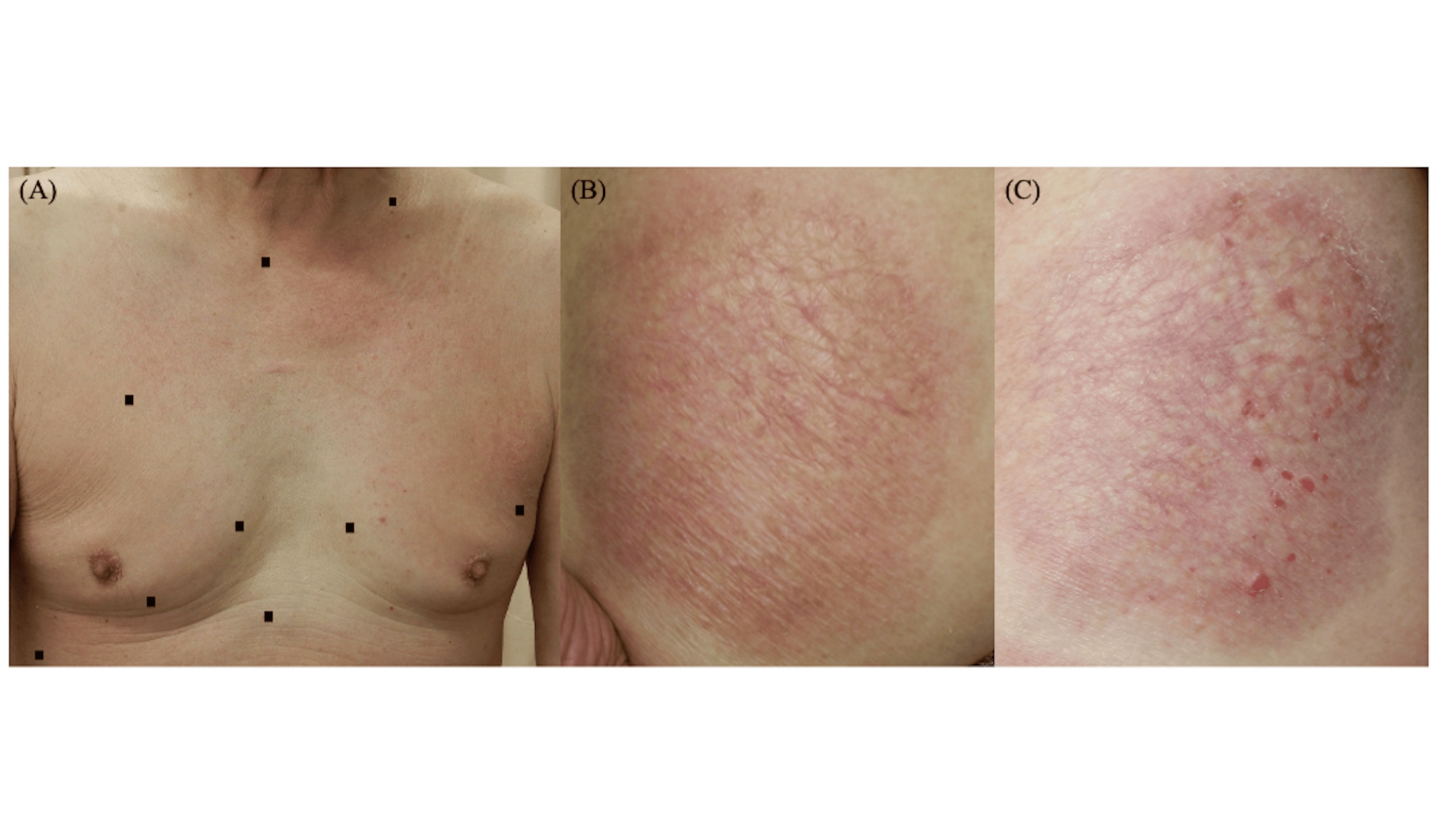
Clinical photographs of the trunk and thighs (A) Clinical presentation of the trunk at the initial visit, demonstrating multiple erythematous lesions with adherent scales. (B) Clinical presentation of the thigh at the initial visit, demonstrating an approximately 20 cm, well-demarcated, atrophic erythematous plaque with fine wrinkling within the lesion. (C) Clinical presentation of the thigh six months after the initial visit, demonstrating newly developed multiple erythematous papules within the pre-existing atrophic erythematous plaque.

A 73-year-old man presented to our department with a five-year history of pruritic eruptions on the trunk and bilateral thighs. Physical examination revealed diffuse, faint erythema with fine scaling on the trunk (Figure 1A) and well-defined, atrophic erythematous plaques about 20 cm in diameter with fine surface wrinkling on both thighs (Figure 1B). There was no evidence of lymphadenopathy, and the patient did not exhibit fever, weight loss, or night sweats. Topical corticosteroid therapy was initiated but produced no remarkable improvement. Approximately six months after the first visit, the erythematous patches with scaling on the trunk had worsened, and new erythematous papules developed within the atrophic plaques on the thighs and on the lower abdomen (Figure 1C). Laboratory investigations, including lactate dehydrogenase, soluble interleukin-2 receptor, and human T-cell leukemia virus type 1 antibody, revealed no abnormalities.

**Figure 2:**
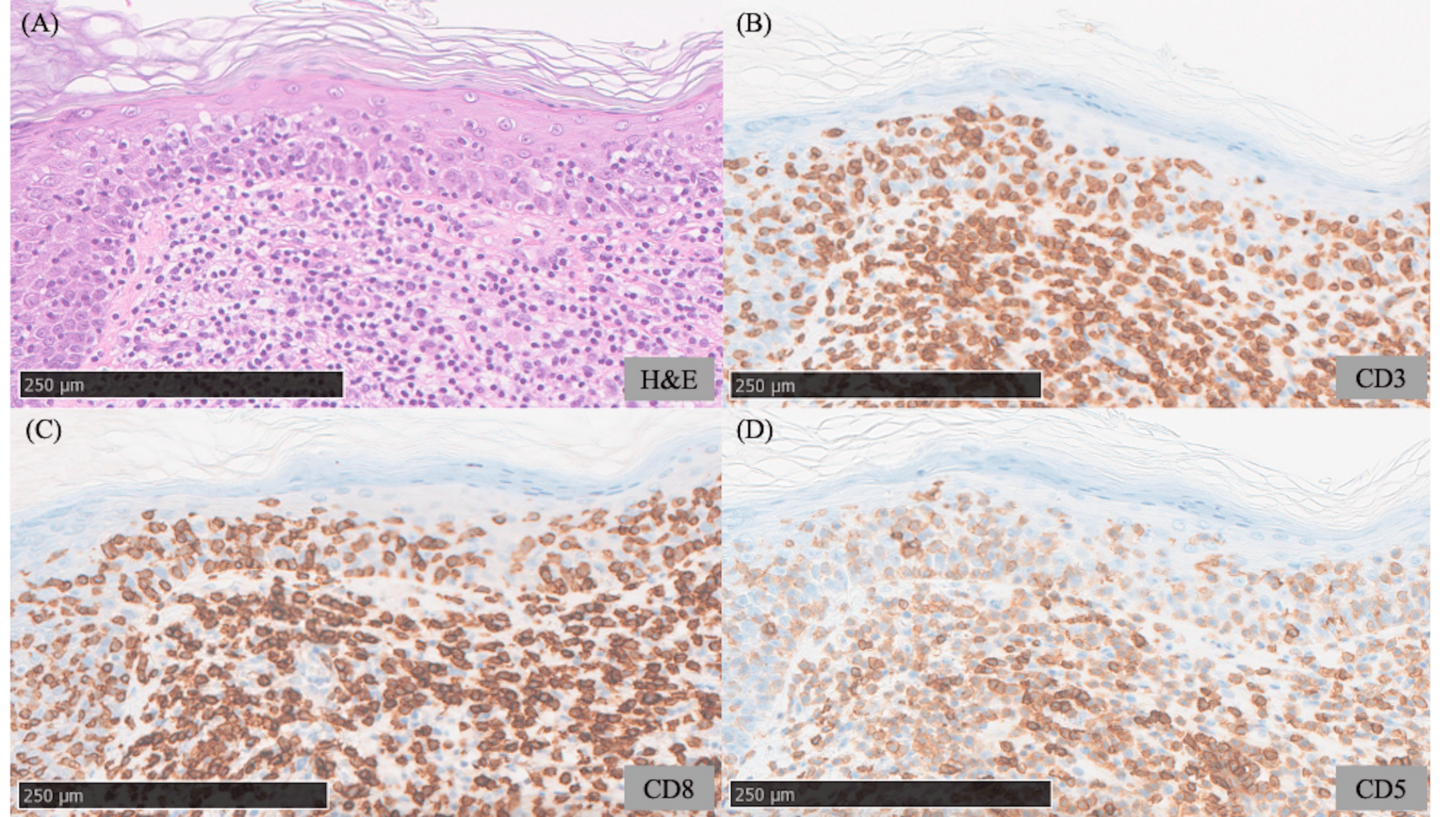
Histopathological findings from a biopsy of the scaly erythematous lesion on the trunk (A) Hematoxylin and eosin staining demonstrates a dense dermal infiltrate of mononuclear cells accompanied by characteristic wiry bundles of collagen, with prominent epidermotropism. Scale bar: 250 μm. (B-D) Immunohistochemical staining for CD3, CD8, and CD5. The infiltrating lymphocytes are positive for CD3 and CD8. CD5-positive cells are relatively sparse compared with CD3-positive cells. Scale bar: 250 μm.

**Figure 3:**
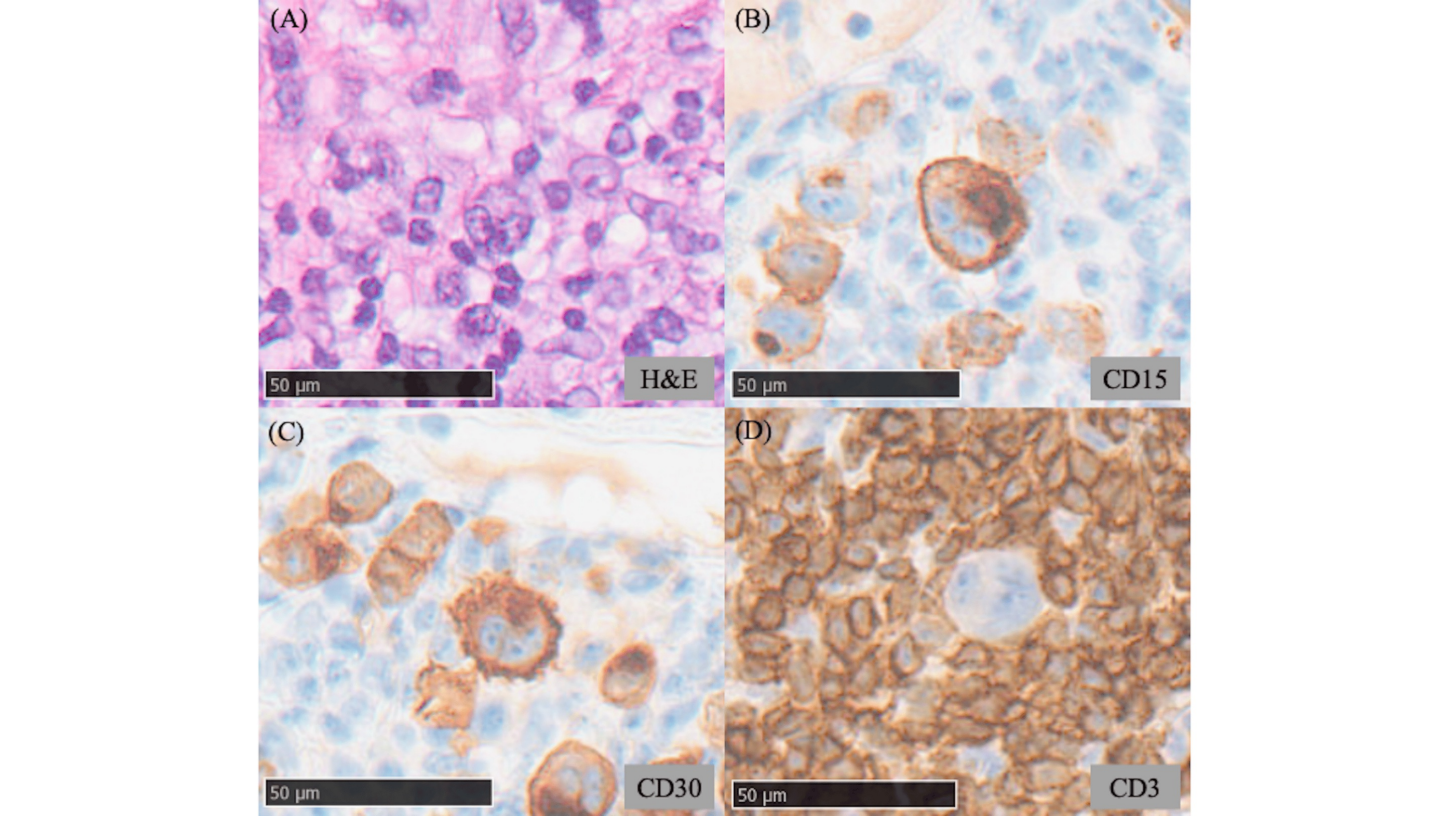
Histopathological features of a biopsy specimen obtained from erythematous papules within an atrophic plaque (A) Hematoxylin and eosin staining reveals Reed-Sternberg-like large atypical cells with prominent nucleoli and multilobated nuclei, admixed with small lymphocytes and histiocytes in the dermal infiltrate. Scale bar: 50 μm. (B-D) Immunohistochemical staining reveals that the Reed-Sternberg-like cells are positive for CD15 and CD30 and are negative for CD3. Scale bar: 50 μm.

A skin biopsy from the scaly erythematous patch on the trunk demonstrated dense infiltration of large atypical lymphocytes in the superficial dermis with prominent epidermotropism (Figure 2A). In addition, wiry bundles of collagen were observed in the dermis. Immunohistochemical examination demonstrated that the infiltrating lymphocytes expressed CD3 and CD8, whereas CD5-positive cells were relatively sparse compared with CD3-positive cells (Figures 2B-2D). A biopsy from the atrophic lesion on the thigh revealed similar findings. Based on the clinical course and histopathological features, the erythematous patches on the trunk and the atrophic plaques on the thighs were diagnosed as CD8-positive MF (T2N0M0B0, Stage IB). A biopsy of the erythematous papule within the atrophic plaque on the thigh demonstrated dense cellular infiltration in the superficial dermis, composed predominantly of small lymphocytes and histiocytes, with occasional eosinophils. Large RS-like cells with lobulated nuclei and prominent nucleoli were also observed (Figure 3A). Immunohistochemistry demonstrated that these large cells were positive for CD15 and CD30 but negative for CD3, CD4, and CD8 (Figures 3B-3D). Such large atypical cells with this immunophenotype were not identified in the biopsy specimens from the MF lesions on the trunk. Flow cytometric analysis of the papular lesion did not detect any abnormal cell populations. Polymerase chain reaction analysis for immunoglobulin gene rearrangement revealed no evidence of clonality. Considering the possibility of cutaneous involvement by HL, positron emission tomography-computed tomography (PET-CT) was performed, but no abnormal uptake was detected in the lymph nodes. Since cutaneous involvement of HL was initially considered, analysis of T-cell receptor gene rearrangement was not performed. At approximately one month of follow-up, the erythematous papules on the thighs and lower abdomen had spontaneously regressed; however, a similar papule subsequently developed near the umbilicus, which also underwent spontaneous regression within two months. Based on the clinical course and pathological findings, the papular lesions were diagnosed as LyP.

For the treatment of MF, oral etretinate (10-20 mg/day, adjusted according to clinical response) and narrowband ultraviolet B phototherapy (initiated at 0.2 J/cm² and gradually increased to 0.7 J/cm², administered every two weeks) were initiated. The scaly erythematous lesions showed gradual improvement, and no recurrence of the erythematous papules has been observed.

## Discussion

In the present case, large atypical cells within MF lesions were negative for CD3, CD4, and CD8 while expressing CD15 and CD30. These cells exhibited RS-like morphology, raising a differential diagnosis of cutaneous involvement by HL, LCT of MF, or LyP arising in association with MF. As the former two entities are associated with poor prognosis [1,4], careful clinical and pathological distinction is essential.

With respect to HL, although RS-like cells typically express both CD15 and CD30, our patient showed no evidence of nodal disease on PET-CT and no aberrant cell populations on flow cytometry suggestive of HL. Moreover, the spontaneous regression of papules observed in this case within one to two months is inconsistent with the clinical course expected in cutaneous involvement of HL, which typically exhibits a tendency to expand.

In MF with LCT, neoplastic cells often express CD30, whereas CD15 positivity is rare. Nevertheless, previous reports have described MF cases showing loss of pan T-cell markers, expression of both CD15 and CD30, and associated lymph node involvement [5]. In such cases, histopathological distinction between MF with LCT and LyP may be challenging. MF with LCT is generally associated with a worse prognosis compared with cases of MF with LyP (five-year overall survival: 63% for MF with LCT vs. 93% for MF with LyP) [1,6]. In the current case, the erythematous papules underwent spontaneous regression, and no tumorous lesions developed at other sites, which is uncharacteristic of MF with LCT.

By contrast, LyP is characterized clinically by recurrent, self-healing papules and histologically by RS-like large atypical cells that express CD30 [2]. Furthermore, previous reports have indicated that CD15 expression is observed in 18% of LyP cases (7 of 39 cases) [3]. In the present case, the occurrence of spontaneously regressing papules within MF lesions, together with the observed immunophenotype, supported the diagnosis of LyP arising in association with MF. Nevertheless, the possibility of MF with LCT cannot be entirely ruled out, and continued vigilant monitoring is considered necessary.

LyP often exhibits morphological features similar to cutaneous involvement of HL and MF with LCT, making comprehensive evaluation, including clinical course and immunophenotype, essential. CD8-positive MF accounts for less than 5% of all MF cases [7], and the coexistence of LyP within MF lesions, as observed in the present case, is considered even rarer. Accumulation of such rare cases is expected to contribute to improved diagnostic accuracy and a deeper understanding of the pathogenesis of cutaneous T-cell lymphomas.

## Conclusions

In the present case, RS-like large atypical cells expressing both CD15 and CD30 were identified within MF lesions. However, based on the clinical course and imaging findings, cutaneous involvement by HL was considered unlikely. Moreover, the spontaneous regression observed in the lesions suggested that MF with LCT was unlikely. Consequently, the diagnosis was established as LyP arising in the setting of MF. Given that LyP can morphologically resemble cutaneous involvement by HL or MF with LCT, an integrated assessment incorporating clinical course, imaging, and histopathological findings is indispensable for accurate diagnosis.
